# Assessing perceptions of nursing knowledge, attitudes, and practices in diabetes management within Chinese healthcare settings

**DOI:** 10.3389/fpubh.2024.1426339

**Published:** 2024-08-12

**Authors:** Lan Hu, Wen Jiang

**Affiliations:** Department of Endocrinology, The Central Hospital of Enshi Tujia and Miao Autonomous Prefecture, Enshi, Hubei, China

**Keywords:** nurses, healthcare workers, KAP, diabetes mellitus, diabetes management

## Abstract

**Background:**

Effective management of diabetes mellitus (DM) involves comprehensive knowledge, attitudes, and practices (KAP) by nurses, which is essential for optimal patient care and aiding patients in their self-management of the condition.

**Method:**

This survey evaluates nurses' self-assessed knowledge, attitudes, and practices (KAP) related to diabetes management, focusing on their perceptions of personnel expertise and care approaches. Using a stratified sampling method, the survey was disseminated across various online platforms from January 2023 to February 2024 within China, including WeChat and Sina Weibo. We employed binary logistic regression and Chi-square tests to explore the statistical correlates of KAP related to DM.

**Results:**

A total of 4,011 nurses participated, revealing significant perceived knowledge deficiencies in specialized DM management areas, with only 34% (*n* = 1,360) proficient in current pharmacological treatments. Attitudinal assessments showed that 54% (*n* = 2,155) recognized the importance of cultural competence in dietary counseling. Practices were strong in routine glucose monitoring (96%, *n* = 3,851) but weaker in psychological support (68%, *n* = 2,736). Regression analysis indicated significant effects of experience on KAP, where nurses with 1–5 years of experience were more likely to show better knowledge (OR = 1.09; *p* = 0.08), and those with advanced degrees demonstrated higher competence (OR = 1.52; *p* = 0.028). Marital status influenced attitudes, with single nurses more likely to exhibit positive attitudes (OR = 0.49; *p* < 0.001), and work environment impacted knowledge, with hospital-based nurses more knowledgeable (OR = 1.15; *p* = 0.14). Additionally, gender differences emerged, with male nurses showing greater knowledge (OR = 1.65; *p* = 0.03) and better practices in diabetes care (OR = 1.47; *p* = 0.04).

**Conclusion:**

The study underscores the critical need for targeted educational programs and policy interventions to enhance nursing competencies in DM management. While the study provides valuable insights into nurses' perceptions of their competencies, future research should incorporate objective knowledge assessments to ensure a comprehensive understanding of their actual capabilities. Interestingly, the data also suggests a substantial opportunity to leverage technology and inter-professional collaboration to further enhance DM management efficacy among nurses, fostering an integrated care approach.

## 1 Introduction

Diabetes mellitus (DM) represents a significant global public health issue, characterized by chronic hyperglycemia resulting from issues in insulin secretion, insulin action, or both (1, 2). The International Diabetes Federation (IDF) estimates that ~537 million adults were living with diabetes in 2021, and this number is expected to rise to 643 million by 2030 (3–5). In China, the prevalence of DM has notably increased in recent decades, now affecting over 114 million adults, which represents ~11% of the adult population (6, 7). This chronic metabolic disorder is characterized by either a lack of insulin production or the body's inability to effectively use insulin, presenting growing difficulties on a global scale and affecting people of all socioeconomic backgrounds (8, 9). It is considered one of the leading causes of illness and death worldwide; alarmingly, in 2015, diabetes was linked to almost 5 million fatalities in persons between the ages of 20 and 79 (10, 11). The increased death rates can be attributed to many consequences, including cerebrovascular diseases, renal failure, eyesight impairment, cardiovascular disorders, and limb amputations (12–14). These negative consequences are worsened by the worldwide trend toward increasingly inactive lifestyles and cultural changes that encourage unhealthy eating habits and reduce levels of physical activity (11). Therefore, it is crucial to prioritize the reduction of these risk factors to decrease the occurrence of DM and enhance public health results (15).

Nurses play a crucial role in the management of diabetes, as they often serve as primary caregivers and are directly involved in patient education and care (16, 17). Holistic management, which encompasses a comprehensive, multifactorial approach to care, is increasingly recognized as crucial in managing diabetes effectively (18). Assessing the KAP of nurses regarding holistic diabetes management is vital for identifying gaps in the current healthcare provision and for developing targeted educational and training programs (18). Studies have shown that enhancing nurses' competencies can lead to improved patient outcomes, direct care, and support in self-management practices in diabetes care (18–21). However, there is limited data available on the KAP among nurses in China regarding the holistic management of DM (22).

Several studies have indicated variability in KAP among nurses concerning diabetes care, often associated with differences in education levels, regional healthcare policies, and available resources (20, 21, 23–26). For instance, research in urban hospitals in Beijing showed higher levels of knowledge and more positive attitudes compared to rural areas, where resources and training opportunities tend to be more limited (27). Understanding these disparities is critical for developing targeted educational programs, funding, and policy interventions aimed at enhancing holistic diabetes care (26). Moreover, given the rapid evolution of diabetes treatment protocols and the integration of technological advances in patient care, continual professional development and training are paramount for nursing staff (22).

Nurses are essential in diabetes management, serving as primary caregivers involved in patient education and care. Holistic management, which includes a comprehensive, multifactorial approach, is crucial for effective diabetes care. In this study, “knowledge” refers to understanding diabetes pathophysiology, treatment protocols, and self-management techniques. “Attitudes” encompass nurses' beliefs and perspectives on diabetes care, including holistic and culturally competent approaches. “Practices” involve implementing diabetes care protocols, patient education, and management strategies. Effective self-management, facilitated by nurses, is vital for optimal glycemic control and complication prevention. However, the extent of nurses' knowledge and skills for holistic diabetes management, particularly in China, remains under-examined. Nurses' attitudes and practices significantly influence their ability to educate and support patients, with China's cultural, systemic, and educational frameworks providing a unique context that may differ from Western settings (22, 28, 29). Given this background, the current study aimed to evaluate nurses' self-perceived knowledge, attitudes, and practices concerning diabetes management within Chinese healthcare institutions. In addition, the perceptions influence their professional behaviors and identify areas for educational improvements.

## 2 Methodology

### 2.1 Study

To investigate the KAP of nurses in the comprehensive management of DM, we conducted a systematic evaluation via a digital, anonymized survey. This cross-sectional study harnessed a stratified sampling method to disseminate the survey hyperlink across multiple online channels, using the snowball technique, including WeChat, Sina Weibo, QQ, email, and other prominent social networks utilized predominantly within China. A total of 5,000 nurses were invited to participate via email and social media platforms. Out of these, 4,011 nurses responded, resulting in a participation rate of 80.22%. Information on those who declined to participate was not systematically recorded. Participants were mandated to respond to each query on the survey, available in both Mandarin and English. The data collection phase extended from January 2023 to February 2024. Participation was voluntary, with nurses informed about the study's aims and assured of the confidentiality and anonymity of their inputs. The study covered diverse geographical locales across China, integrating both urban and rural healthcare settings. Data anonymization was ensured by assigning unique codes to each participant and removing any identifying information before analysis.

### 2.2 Inclusion and exclusion criteria

Inclusion criteria were as follows: (i) registered nurses currently practicing in China, and (ii) age 18 years or older. These criteria remained consistent throughout the study. Exclusion criteria included (i) nurses with diagnosed DM, as their personal experiences might skew perceptions, (ii) non-resident nurses temporarily working in China, and (iii) those unable to provide informed consent due to any reason.

### 2.3 Measurements

The survey tool was meticulously crafted by modifying and integrating elements from previously validated instruments pertinent to diabetes care. The cut-off of ~50% was set following precedents in previous studies, ensuring consistency and comparability of results across similar research (20, 21, 23–26, 30–32). We assessed nurses' self-perceived knowledge through a series of 16 questions covering topics such as the pathophysiology of DM, current treatment protocols, patient education strategies, and self-management techniques. These questions were designed to gauge how confident nurses felt about their knowledge rather than objectively measuring their actual knowledge. Each correct response was awarded one point, with a total possible score of 16. A threshold of ≥50% (eight points) was set to classify respondents as knowledgeable.

Furthermore, we evaluated attitudes toward DM management using 14 questions about personal beliefs, perceived efficacy of treatment modalities, and readiness to implement holistic care approaches. Attitude scores were allocated based on responses, with “Agree” scoring one point, reflecting a positive orientation, and “Disagree” or “Uncertain” scoring zero. A cutoff of seven was used to differentiate between predominantly positive and negative attitudes.

In addition, practices were scrutinized using eight questions related to the implementation of diabetes management protocols, participation in diabetes education programs, and adherence to clinical guidelines. Practice scores were calculated by assigning one point for each affirmative response indicative of best practices. The cutoff score of five for practice behaviors was determined based on prior studies and expert consultations.

Responses of “Not Sure” were categorized as “No” for knowledge, attitudes, and practices. This conservative approach ensured that only confident affirmative responses were considered as “Yes”, maintaining the study's rigor by treating uncertainty as a lack of knowledge or negative attitude. Academic qualifications were categorized as follows: Associate Degree in Nursing (typically 2–3 years of study), Bachelor of Science in Nursing (4 years), Master of Science in Nursing (two additional years post-bachelor), and Doctor of Nursing Practice (3–4 additional years post-master). The survey further collected detailed demographic information to explore correlations between these factors and KAP results. Specific questions included years of nursing experience, gender, marital status, type of institution employed in (hospital, clinic), the highest level of educational attainment, employment status (full-time, part-time, other), and primary department of work within the healthcare facility.

### 2.4 Statistical analysis

In this study, the statistical analysis was rigorously conducted using both exploratory and inferential techniques to comprehensively evaluate the data gathered from the survey on DM management among nurses. The initial step involved summarizing the frequency distributions of socio-demographic variables via descriptive statistical analysis. All statistical computations were executed using R statistical software (version 4.2.2). We managed our datasets using the R environment, leveraging several packages such as dplyr for data manipulation and ggplot2 for graphical representations. Instead of using traditional reliability measures like Cronbach's alpha, we opted for Guttman's λ^2^, which we calculated to be 0.86, suggesting the high reliability of the survey instrument without the stringent assumptions required by Cronbach's alpha (33). Further, binary logistic regression was employed to ascertain the Odds Ratio (ORs), which provided insights into the likelihood of high knowledge, positive attitudes, and effective practices among the nurses based on predictor variables. This was complemented with the reporting of regression coefficients, their significance levels, and 95% confidence intervals (CI) to reinforce the robustness of our findings.

## 3 Results

### 3.1 Social and demographic characteristics

In the current study, a total of 4,011 registered nurses participated, yielding significant insights into their demographics and professional backgrounds. The distribution of experience notably concentrates in the 1–5 years category with 55% (*n* = 2,205), while a large majority, 71% (*n* = 2,867), of the cohort are female, and 61% (*n* = 2,436) reported being single. Work settings are heavily skewed toward hospitals at 71% (*n* = 2,839), with 61% (*n* = 2,448) holding an Associate Degree in Nursing. Employment is nearly equally divided between contract positions at 47% (*n* = 1,870) and permanent roles at 48% (*n* = 1,930). Nurses are primarily deployed in emergency rooms (43%, *n* = 1,742) and general medicine (32%, *n* = 1,294). In terms of professional competency in DM management, 74% (*n* = 2,971) of nurses are classified as knowledgeable. Attitudes toward their practice are positive for 84% (*n* = 3,362), and a substantial 93% (*n* = 3,719) engage in good practice. These data points underscore crucial areas for targeted educational and policy initiatives to boost the efficacy of diabetes care (Table 1).

**Table 1:** Demographics of study participants.

**Variable**	*n* = 4,011^*^
**How many years of experience do you have in nursing?**	
< 1 year	965 (24%)
1–5 years	2,205 (55%)
11–15 years	24 (0.6%)
6–10 years	778 (19%)
More than 15 years	39 (1.0%)
**What is your gender?**	
Female	2,867 (71%)
Male	1,144 (29%)
**What is your marital status?**	
Divorced	96 (2.4%)
Married	1,478 (37%)
Single	2,436 (61%)
Widowed	1 (< 0.1%)
**What type of institution do you currently work in?**	
Clinic	166 (4.1%)
Hospital	2,839 (71%)
Private practice	1,006 (25%)
**What is your highest level of education?**	
Associate Degree in Nursing	2,448 (61%)
Bachelor of Science in Nursing	464 (12%)
Doctor of Nursing Practice	313 (7.8%)
Master of Science in Nursing	786 (20%)
**What is your employment status?**	
Contract	1,870 (47%)
Permanent employee	1,930 (48%)
Visiting	211 (5.3%)
**In which department do you primarily work?**	
Emergency room	1,742 (43%)
Endocrinology	443 (11%)
General medicine	1,294 (32%)
Intensive care unit	299 (7.5%)
Other	233 (5.8%)
**Knowledge**	
Knowledgeable	2,971 (74%)
Not knowledgeable	1,040 (26%)
**Attitude**	
Negative attitude	649 (16%)
Positive attitude	3,362 (84%)
**Practice**	
Good practice	3,719 (93%)
Bad practice	292 (7.3%)

^*^1 *n* (%).

### 3.2 Knowledge assessment

**Figure 1:**
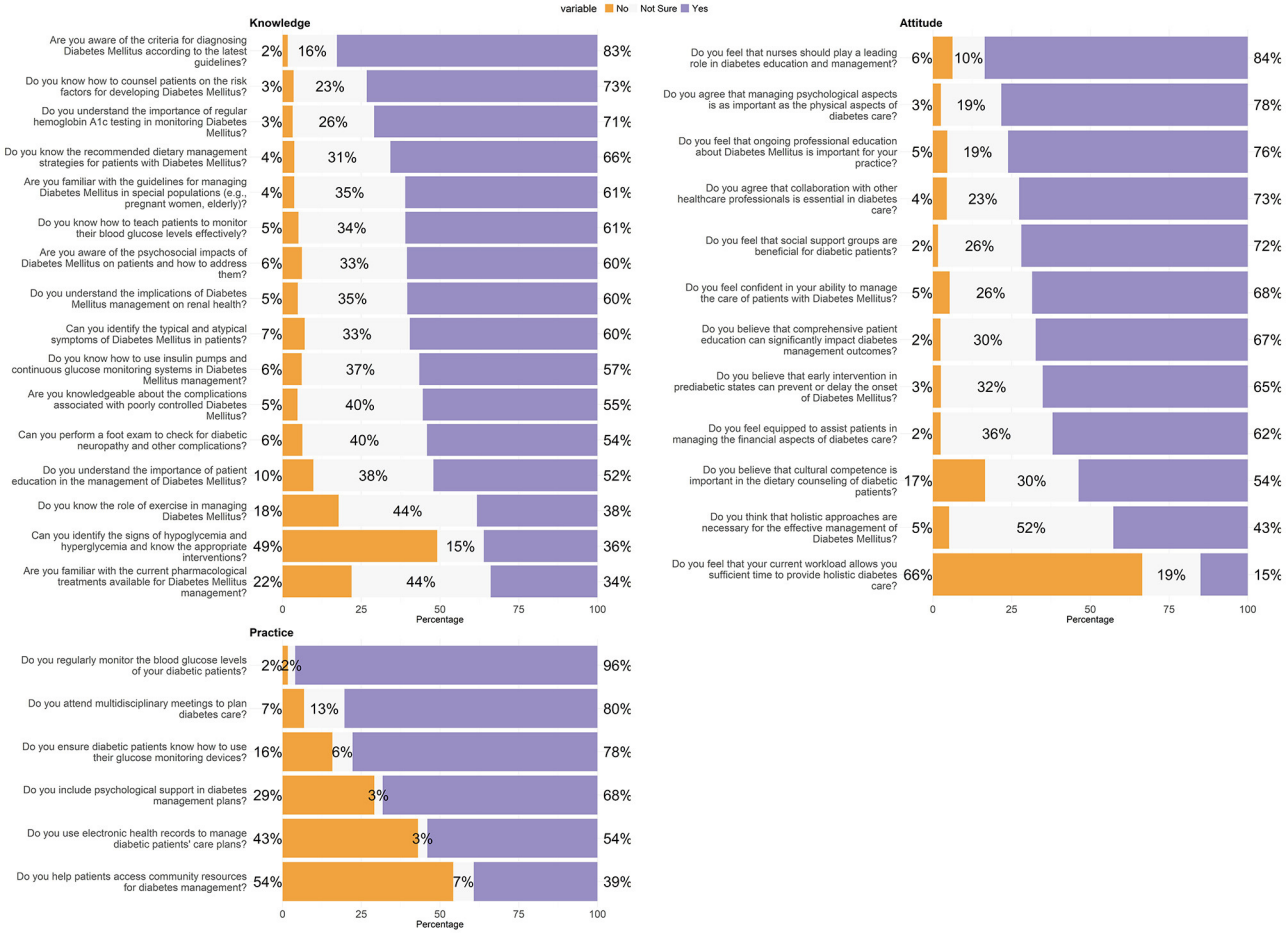
Nurses' knowledge, attitudes, and practices on diabetes management.

Table 2 provides a comprehensive evaluation of the knowledge level among participants regarding various aspects of DM management. The data highlights areas where knowledge is well-established and areas where improvement is needed. The vast majority of participants (83%, *n* = 3,320) are familiar with the criteria for diagnosing DM according to the latest guidelines, demonstrating a strong awareness of foundational diagnostic criteria. However, knowledge gaps appear in more specialized areas, such as the management of DM in special populations like pregnant women and the older adults, where only 61% (*n* = 2,448) feel confident. Similarly, while a majority understands the psychosocial impacts of DM (60%, *n* = 2,426) and complications associated with poor control (55%, *n* = 2,225), there is less certainty about current pharmacological treatments, with only 34% (*n* = 1,360) indicating familiarity. A significant concern is the high percentage of respondents who lack confidence in identifying the signs of hypoglycemia and hyperglycemia (49%, *n* = 1,969), which are critical skills for effective patient management. This highlights a crucial need for enhanced training and education. Responses also show that 73% (*n* = 2,939) of nurses are adept at counseling patients on risk factors, and a comparable majority (61%, *n* = 2,447) can teach patients to monitor their blood glucose levels effectively. Knowledge about the use of technology in management, like insulin pumps and continuous glucose monitoring systems, is moderately high at 57% (*n* = 2,269). The role of lifestyle factors in managing DM is less well-understood; only 38% (*n* = 1,535) are familiar with the role of exercise, indicating a potential area for educational interventions, as shown in Figure 1.

**Table 2:** Knowledge assessment of participants.

	**Responses**		
	**No**	**Not sure**	**Yes**
**Statement**			
Are you aware of the criteria for diagnosing diabetes mellitus according to the latest guidelines?	65 (1.6%)	626 (16%)	3,320 (83%)
Are you aware of the psychosocial impacts of diabetes mellitus on patients and how to address them?	247 (6.2%)	1,338 (33%)	2,426 (60%)
Are you familiar with the current pharmacological treatments available for diabetes mellitus management?	880 (22%)	1,771 (44%)	1,360 (34%)
Are you familiar with the guidelines for managing diabetes mellitus in special populations (e.g., pregnant women, older adults)?	148 (3.7%)	1,415 (35%)	2,448 (61%)
Are you knowledgeable about the complications associated with poorly controlled diabetes mellitus?	190 (4.7%)	1,596 (40%)	2,225 (55%)
Can you identify the signs of hypoglycemia and hyperglycemia and know the appropriate interventions?	1,969 (49%)	594 (15%)	1,448 (36%)
Can you identify the typical and atypical symptoms of diabetes mellitus in patients?	282 (7.0%)	1,338 (33%)	2,391 (60%)
Can you perform a foot exam to check for diabetic neuropathy and other complications?	253 (6.3%)	1,587 (40%)	2,171 (54%)
Do you know how to counsel patients on the risk factors for developing diabetes mellitus?	140 (3.5%)	932 (23%)	2,939 (73%)
Do you know how to teach patients to monitor their blood glucose levels effectively?	203 (5.1%)	1,361 (34%)	2,447 (61%)
Do you know how to use insulin pumps and continuous glucose monitoring systems in diabetes mellitus management?	244 (6.1%)	1,498 (37%)	2,269 (57%)
Do you know the recommended dietary management strategies for patients with diabetes mellitus?	150 (3.7%)	1,224 (31%)	2,637 (66%)
Do you know the role of exercise in managing diabetes mellitus?	714 (18%)	1,762 (44%)	1,535 (38%)
Do you understand the implications of diabetes mellitus management on renal health?	194 (4.8%)	1,395 (35%)	2,422 (60%)
Do you understand the importance of patient education in the management of diabetes mellitus?	392 (9.8%)	1,530 (38%)	2,089 (52%)
Do you understand the importance of regular hemoglobin A1c testing in monitoring diabetes mellitus?	129 (3.2%)	1,037 (26%)	2,845 (71%)
Total	6,200 (9.7%)	21,004 (33%)	36,972 (58%)

### 3.3 Attitude assessment

Table 3 delineates the attitudes of participants toward various facets of diabetes care, highlighting the consensus and areas of ambivalence among the nursing professionals surveyed. A significant majority of respondents, 73% (*n* = 2,912), recognize the essential nature of collaboration with other healthcare professionals in diabetes care, reflecting a widespread appreciation for interdisciplinary approaches.

**Table 3:** Attitude assessment of participants.

	**Responses**		
	**No**	**Not sure**	**Yes**
**Statement**			
Do you agree that collaboration with other healthcare professionals is essential in diabetes care?	178 (4.4%)	921 (23%)	2,912 (73%)
Do you agree that managing psychological aspects is as important as the physical aspects of diabetes care?	102 (2.5%)	770 (19%)	3,139 (78%)
Do you believe that comprehensive patient education can significantly impact diabetes management outcomes?	98 (2.4%)	1,211 (30%)	2,702 (67%)
Do you believe that cultural competence is important in the dietary counseling of diabetic patients?	665 (17%)	1,191 (30%)	2,155 (54%)
Do you believe that early intervention in prediabetic states can prevent or delay the onset of diabetes mellitus?	102 (2.5%)	1,297 (32%)	2,612 (65%)
Do you feel confident in your ability to manage the care of patients with diabetes mellitus?	214 (5.3%)	1,050 (26%)	2,747 (68%)
Do you feel equipped to assist patients in managing the financial aspects of diabetes care?	99 (2.5%)	1,426 (36%)	2,486 (62%)
Do you feel that nurses should play a leading role in diabetes education and management?	250 (6.2%)	410 (10%)	3,351 (84%)
Do you feel that ongoing professional education about diabetes mellitus is important for your practice?	184 (4.6%)	774 (19%)	3,053 (76%)
Do you feel that social support groups are beneficial for diabetic patients?	66 (1.6%)	1,061 (26%)	2,884 (72%)
Do you feel that your current workload allows you sufficient time to provide holistic diabetes care?	2,666 (66%)	744 (19%)	601 (15%)
Do you think that holistic approaches are necessary for the effective management of diabetes mellitus?	208 (5.2%)	2,091 (52%)	1,712 (43%)
Total	4,832 (10%)	12,946 (27%)	30,354 (63%)

Similarly, 78% (*n* = 3,139) agree that managing psychological aspects is as important as addressing the physical aspects of diabetes care, indicating a holistic understanding of patient needs. This is further supported by the 67% (*n* = 2,702) who believe that complete patient education can significantly influence diabetes management outcomes, emphasizing the role of education in effective diabetes care. However, attitudes vary concerning cultural competence in dietary counseling, with only 54% (*n* = 2,155) acknowledging its importance, suggesting a potential area for further training and awareness. Another notable insight is the strong endorsement of early intervention in prediabetic states to prevent or delay the onset of DM, supported by 65% (*n* = 2,612) of participants.

The confidence in managing diabetes care is affirmed by 68% (*n* = 2,747) of the nurses. However, only 62% (*n* = 2,486) feel equipped to assist patients in managing the financial aspects of their care, indicating a gap in addressing the economic challenges faced by patients. A substantial 84% (*n* = 3,351) feel that nurses should play a leading role in diabetes education and management, and 76% (*n* = 3,053) recognize the importance of ongoing professional education in their practice. This is congruent with the 72% (*n* = 2,884) who see social support groups as beneficial for diabetic patients, underscoring the value placed on community and continuing education. Contrastingly, a significant majority, 66% (*n* = 2,666), feel that their current workload does not allow them sufficient time to provide holistic diabetes care, highlighting systemic constraints that may hinder the optimal delivery of care. Moreover, the necessity of holistic approaches in diabetes management garners less consensus, with only 43% (*n* = 1,712) endorsing this perspective amidst a substantial 52% (*n* = 2,091) unsure, suggesting an area ripe for further exploration and advocacy in the professional community, as shown in Figure 1.

### 3.4 Practice assessment

Table 4 assesses the practical engagement of participants in the management of diabetes care, revealing strong adherence to several best practices while identifying areas where improvement could be beneficial. Most nurses, 80% (*n* = 3,222), actively participate in multidisciplinary meetings to plan diabetes care, illustrating a robust collaborative practice among healthcare professionals.

**Table 4:** Practice assessment of participants.

	**Responses**		
	**No**	**Not sure**	**Yes**
**Statement**			
Do you attend multidisciplinary meetings to plan diabetes care?	274 (6.8%)	515 (13%)	3,222 (80%)
Do you ensure diabetic patients know how to use their glucose monitoring devices?	635 (16%)	256 (6.4%)	3,120 (78%)
Do you help patients access community resources for diabetes management?	2,173 (54%)	263 (6.6%)	1,575 (39%)
Do you include psychological support in diabetes management plans?	1,169 (29%)	106 (2.6%)	2,736 (68%)
Do you regularly monitor the blood glucose levels of your diabetic patients?	67 (1.7%)	93 (2.3%)	3,851 (96%)
Do you use electronic health records to manage diabetic patients' care plans?	1,724 (43%)	120 (3.0%)	2,167 (54%)
Total	6,042 (25%)	1,353 (5.6%)	16,671 (69%)

Moreover, a significant 78% (*n* = 3,120) ensure that diabetic patients are proficient in using their glucose monitoring devices, which is crucial for the day-to-day management of their condition. Similarly, the monitoring of blood glucose levels is nearly universal among respondents, with 96% (*n* = 3,851) regularly performing this essential task, reflecting a high level of diligence in patient care. However, the responses also highlight some areas needing attention. Although 68% (*n* = 2,736) include psychological support in their management plans, indicating a holistic approach to patient care, there remains a significant portion of nurses who could further integrate this critical aspect into their routines. The use of electronic health records (EHRs) to manage care plans is reported by 54% (*n* = 2,167) of the participants, suggesting that there is room for increased adoption of this technology to enhance patient management efficiency. Additionally, helping patients access community resources for diabetes management appears to be a less frequent practice, with only 39% (*n* = 1,575) actively assisting in this area, indicating a potential gap in fully supporting patients beyond clinical settings, as shown in Figure 1.

### 3.5 Binary logistic regression

#### 3.5.1 Statistical correlates of knowledge level in nursing professionals

The dataset comprises 4,011 participants, categorized into “Knowledgeable” nurses (*n* = 2,971) and “Not Knowledgeable” (*n* = 1,040) nurses. Statistical analyses, including Chi-square tests and binary logistic regression, were utilized to examine the influence of various variables on knowledge levels.

In terms of years of nursing experience, significant differences in knowledge levels were observed (Chi-square *p* < 0.001). Notably, nurses with more than 15 years of experience showed a significantly lower likelihood of being knowledgeable OR = 0.08, 95% CI = 0.01–0.27, *p* < 0.001). Conversely, those with 1–5 years of experience exhibited a moderately higher knowledge level, though this was not statistically significant (OR = 1.09, 95% CI = 0.87–1.36, *p* = 0.08). Regarding demographic variables, marital status revealed significant effects (Chi-square *p* < 0.001), with single nurses being more likely to be knowledgeable compared to their married or divorced counterparts (OR = 0.49, 95% CI = 0.30–0.81, *p* < 0.001 for singles). The work environment also impacted knowledge levels significantly (Chi-square *p* < 0.001). Nurses employed in hospitals were somewhat more likely to be knowledgeable (OR = 1.15, 95% CI = 0.80–1.69, *p* = 0.14), whereas those in private practice were less likely to be knowledgeable (OR = 0.72, 95% CI = 0.49–1.09, *p* < 0.001). Educational attainment strongly correlated with knowledge, where nurses with a Doctor of Nursing Practice were significantly more likely to be knowledgeable (OR = 0.23, 95% CI = 0.15–0.33, *p* < 0.001). However, the attitude toward nursing practice significantly differentiated knowledge levels (Chi-square *p* < 0.001). Nurses with a positive attitude were twice as likely to be knowledgeable compared to those with a negative outlook (OR = 0.43, 95% CI = 0.36–0.52, *p* < 0.001). Overall, these results underscore the complex interplay of experience, demographic factors, work environment, educational background, and attitude in shaping knowledge levels among nurses (Table 5).

**Table 5:** Binary logistic regression of knowledge among nurses.

**Variable**	***N***	**Knowledgeable, *N* = 2,971**	**Not-knowledgeable, *N* = 1,040**	**Chi-square *P*-value**	**Binary logistic regression analysis**			
				***p*-value**	**Coefficient**	**Odd ratio**	**95% CI**	***p*-value**
**How many years of experience do you have in nursing?**	4,011			< 0.001				< 0.001
< 1 year		756 (25%)	209 (20%)		—	—	—	
1–5 years		1,598 (54%)	607 (58%)		0.08	1.09	0.87, 1.36	
11–15 years		20 (0.7%)	4 (0.4%)		−0.74	0.48	0.13, 1.39	
6–10 years		560 (19%)	218 (21%)		−0.02	0.98	0.73, 1.33	
More than 15 years		37 (1.2%)	2 (0.2%)		−2.6	0.08	0.01, 0.27	
**What is your gender?**	4,011			0.6				0.9
Female		2,131 (72%)	736 (71%)		—	—	—	
Male		840 (28%)	304 (29%)		0.01	1.01	0.86, 1.19	
**What is your marital status?**	4,011			< 0.001				< 0.001
Divorced		58 (2.0%)	38 (3.7%)		—	—	—	
Married		1,065 (36%)	413 (40%)		−0.35	0.7	0.45, 1.12	
Single		1,848 (62%)	588 (57%)		−0.71	0.49	0.30, 0.81	
Widowed		0 (0%)	1 (< 0.1%)		13	596,357	0.00, NA	
**What type of institution do you currently work in?**	4,011			< 0.001				< 0.001
Clinic		123 (4.1%)	43 (4.1%)		—	—	—	
Hospital		2,053 (69%)	786 (76%)		0.14	1.15	0.80, 1.69	
Private practice		795 (27%)	211 (20%)		−0.32	0.72	0.49, 1.09	
**What is your highest level of education?**	4,011			< 0.001				< 0.001
Associate Degree in Nursing		1,747 (59%)	701 (67%)		—	—	—	
Bachelor of Science in Nursing		345 (12%)	119 (11%)		−0.24	0.78	0.61, 1.00	
Doctor of Nursing Practice		283 (9.5%)	30 (2.9%)		−1.5	0.23	0.15, 0.33	
Master of Science in Nursing		596 (20%)	190 (18%)		−0.28	0.76	0.62, 0.93	
**What is your employment status?**	4,011			< 0.001				< 0.001
Contract		1,327 (45%)	543 (52%)		—	—	—	
Permanent employee		1,495 (50%)	435 (42%)		−0.47	0.62	0.52, 0.75	
Visiting		149 (5.0%)	62 (6.0%)		0.03	1.03	0.73, 1.45	
**In which department do you primarily work?**	4,011			< 0.001				0.003
Emergency room		1,315 (44%)	427 (41%)		—	—	—	
Endocrinology		322 (11%)	121 (12%)		−0.04	0.96	0.74, 1.23	
General medicine		930 (31%)	364 (35%)		0.21	1.23	0.99, 1.54	
Intensive care unit		209 (7.0%)	90 (8.7%)		0.11	1.12	0.83, 1.49	
Other		195 (6.6%)	38 (3.7%)		−0.56	0.57	0.39, 0.82	
**Attitude**	4,011			< 0.001				< 0.001
Negative attitude		375 (13%)	274 (26%)		—	—	—	
Positive attitude		2,596 (87%)	766 (74%)		−0.84	0.43	0.36, 0.52	
**Practice**	4,011			0.14				0.4
Good practice		2,744 (92%)	975 (94%)		—	—	—	
Bad practice		227 (7.6%)	65 (6.3%)		−0.12	0.89	0.66, 1.18	

#### 3.5.2 Statistical correlates of attitude in nursing professionals

This analysis encompasses data from 4,011 nursing professionals, stratified into “Negative Attitude” (*n* = 649) and “Positive Attitude” (*n* = 3,362) groups. The influence of various professional and demographic variables on attitudes was assessed using Chi-square tests and binary logistic regression models, yielding significant findings (Chi-square *p* < 0.001 across multiple variables).

Experience in nursing emerged as a significant determinant of attitude (Chi-square *p* < 0.001). Nurses with < 1 year of experience displayed a lower incidence of positive attitudes compared to those with more experience. In contrast, nurses with more than 15 years of experience had markedly higher odds of possessing a positive attitude (OR = 5.79, 95% CI = 2.66–12.4, *p* < 0.001), highlighting the potential impact of extensive professional experience on attitude formation. Gender differences were also pronounced, with males showing higher odds of having a positive attitude compared to females (OR = 2.18, 95% CI = 1.80–2.64, *p* < 0.001). Marital status further influenced attitudes, with single nurses more likely to exhibit a positive attitude compared to their married or divorced peers (OR for singles = 0.19, 95% CI = 0.11–0.33, *p* < 0.001). However, in work settings, nurses employed in hospitals and private practices had lower odds of possessing a positive attitude compared to those in clinics, though the effect was moderate (OR for hospitals = 0.6, 95% CI = 0.41–0.89, *p* = 0.03).

Educational attainment was a strong predictor of attitude. Nurses with a Master of Science in Nursing were significantly less likely to have a positive attitude compared to those with lower educational qualifications (OR = 0.17, 95% CI = 0.12–0.24, *p* < 0.001). Permanent employees and those working in general medicine had slightly lower odds of a positive attitude (OR for permanent employees = 0.94, 95% CI = 0.76–1.17, *p* = 0.8). Knowledge and practice were also significant, with knowledgeable and well-practicing nurses more likely to hold positive attitudes (OR for knowledgeable = 2.36, 95% CI = 1.95–2.86, *p* < 0.001 for knowledge; OR for good practice = 0.57, 95% CI = 0.37–0.85, *p* = 0.005 for practice). These results underscore the multifaceted nature of attitude formation among nurses, influenced by professional experience, demographic traits, educational background and workplace environment influence professional practices, as shown in Table 6.

**Table 6:** Binary logistic regression of attitude in nurses.

**Variable**	***N***	**Negative attitude, *N* = 649**	**Positive attitude, *N* = 3,362**	**Chi-square *P*-value**	**Binary logistic regression analysis**			
				***p*-value**	**Coefficient**	**Odd ratio**	**95% CI**	***p*-value**
**How many years of experience do you have in nursing?**	4,011							< 0.001
< 1 year		95 (15%)	870 (26%)		—	—	—	
1–5 years		442 (68%)	1,763 (52%)		1.1	3.06	2.33, 4.06	
6–10 years		91 (14%)	687 (20%)		0.5	1.65	1.11, 2.45	
11–15 years		5 (0.8%)	19 (0.6%)		0.61	1.84	0.56, 5.13	
More than 15 years		16 (2.5%)	23 (0.7%)		1.8	5.79	2.66, 12.4	
**What is your gender?**	4,011			< 0.001				< 0.001
Female		379 (58%)	2,488 (74%)		—	—	—	
Male		270 (42%)	874 (26%)		0.78	2.18	1.80, 2.64	
**What is your marital status?**	4,011			< 0.001				< 0.001
Divorced		31 (4.8%)	65 (1.9%)		—	—	—	
Married		226 (35%)	1,252 (37%)		−1.4	0.25	0.15, 0.42	
Single		392 (60%)	2,044 (61%)		−1.7	0.19	0.11, 0.33	
Widowed		0 (0%)	1 (< 0.1%)		−13	0		
**What type of institution do you currently work in?**	4,011			< 0.001				0.03
Clinic		44 (6.8%)	122 (3.6%)		—	—	—	
Hospital		462 (71%)	2,377 (71%)		−0.51	0.6	0.41, 0.89	
Private practice		143 (22%)	863 (26%)		−0.57	0.57	0.37, 0.87	
**What is your highest level of education?**	4,011			< 0.001				< 0.001
Associate Degree in Nursing		499 (77%)	1,949 (58%)		—	—	—	
Bachelor of Science in Nursing		64 (9.9%)	400 (12%)		−0.64	0.53	0.39, 0.71	
Doctor of Nursing Practice		43 (6.6%)	270 (8.0%)		−0.64	0.53	0.36, 0.75	
Master of Science in Nursing		43 (6.6%)	743 (22%)		−1.8	0.17	0.12, 0.24	
**What is your employment status?**	4,011			< 0.001				0.8
Contract		359 (55%)	1,511 (45%)		—	—	—	
Permanent employee		264 (41%)	1,666 (50%)		−0.06	0.94	0.76, 1.17	
Visiting		26 (4.0%)	185 (5.5%)		−0.14	0.87	0.54, 1.37	
**In which department do you primarily work?**	4,011			0.2				0.055
Emergency room		284 (44%)	1,458 (43%)		—	—	—	
Endocrinology		79 (12%)	364 (11%)		−0.05	0.95	0.70, 1.29	
General medicine		189 (29%)	1,105 (33%)		−0.26	0.77	0.59, 1.00	
Intensive care unit		51 (7.9%)	248 (7.4%)		−0.14	0.87	0.61, 1.24	
Other		46 (7.1%)	187 (5.6%)		0.36	1.44	0.98, 2.07	
**Knowledge**	4,011			< 0.001				< 0.001
Knowledgeable		375 (58%)	2,596 (77%)		—	—	—	
Not knowledgeable		274 (42%)	766 (23%)		0.86	2.36	1.95, 2.86	
**Practice**	4,011			0.001				0.005
Good practice		621 (96%)	3,098 (92%)		—	—	—	
Bad practice		28 (4.3%)	264 (7.9%)		−0.56	0.57	0.37, 0.85	

#### 3.5.3 Statistical correlates of practice variations in nursing professionals

In the present investigation, we scrutinized a dataset comprising 4,011 nursing professionals to delineate the association between various professional and demographic variables and their adherence to established practice norms, categorically differentiated into “Good Practice” (*n* = 3,719) and “Bad Practice” (*n* = 292). Utilizing Chi-square tests and binary logistic regression analyses, significant statistical correlations were unearthed that elucidate the influence of these variables on nursing practices.

Professional tenure emerged as a salient variable, albeit with nuanced disparities in its influence on practice outcomes. Specifically, nurses with an intermediate level of experience (6–10 years) exhibited a marginally enhanced propensity toward adhering to good practice standards (OR = 1.35, 95% CI = 0.80–2.29, *p* = 0.3). However, this association did not achieve statistical significance. Marital status demonstrated a moderate correlation with practice quality, particularly among single nurses who exhibited an increased likelihood of engaging in good practice (OR = 1.53, 95% CI = 0.62–4.63, *p* = 0.3), though these findings lacked statistical significance. The type of healthcare institution also played a pivotal role, with nurses employed in hospitals demonstrating a higher likelihood of maintaining good practice standards compared to their counterparts in private practices or clinics (OR = 2.15, 95% CI = 1.02–5.55, *p* = 0.048).

Nurses holding a Bachelor of Science in Nursing degree were significantly more inclined to exhibit good practice behaviors compared to those with alternative qualifications (OR = 1.52, 95% CI = 1.04–2.17, *p* = 0.028), underscoring the impact of advanced educational credentials on practice quality. Conversely, employment status exhibited no discernible effect on practice quality, indicating that the contractual nature of employment—whether permanent, contractual, or visiting—did not distinctively influence practice outcomes (OR for permanent employees = 1.14, 95% CI = 0.84–1.54, *p* = 0.6). Departmental affiliation provided additional insights, with nurses in the Endocrinology department more likely to adhere to good practice standards compared to those in other departments (OR = 0.61, 95% CI = 0.38–0.96, *p* = 0.083), suggesting that specialization may exert an influence on practice behaviors. Professional attitude toward the nursing role had a definitive impact, with individuals harboring a positive attitude significantly more inclined toward good practices (OR = 1.81, 95% CI = 1.22–2.80, *p* = 0.003). These findings articulate a complex interplay of factors such as experience, marital status, institutional context, educational background, and professional attitude in modulating nursing practices, which are pivotal for the delivery of efficacious patient care (Table 7).

**Table 7:** Binary logistic regression of practices in nurses.

**Variable**	***N***	**Good practice, *N* = 3,719**	**Bad practice, *N* = 292**	**Chi-square *P*-value**	**Binary logistic regression analysis**			
				***p*-value**	**Coefficient**	**Odd ratio**	**95% CI**	***p*-value**
**How many years of experience do you have in nursing?**	4,011			0.5				0.5
< 1 year		886 (24%)	79 (27%)		—	—	—	
1–5 years		2,045 (55%)	160 (55%)		0.13	1.13	0.80, 1.62	
6–10 years		727 (20%)	51 (17%)		0.3	1.35	0.80, 2.29	
11–15 years		24 (0.6%)	0 (0%)		−12	0	0.00, 34.2	
More than 15 years		37 (1.0%)	2 (0.7%)		0.35	1.42	0.22, 5.28	
What is your gender?	4,011			0.8				>0.9
Female		2,660 (72%)	207 (71%)		—	—	—	
Male		1,059 (28%)	85 (29%)		0.01	1.01	0.76, 1.32	
**What is your marital status?**	4,011			0.007				0.3
Divorced		91 (2.4%)	5 (1.7%)		—	—	—	
Married		1,395 (38%)	83 (28%)		0.05	1.05	0.45, 3.07	
Single		2,232 (60%)	204 (70%)		0.42	1.53	0.62, 4.63	
Widowed		1 (< 0.1%)	0 (0%)		−12	0		
**What type of institution do you currently work in?**	4,011			0.015				0.048
Clinic		160 (4.3%)	6 (2.1%)		—	—	—	
Hospital		2,612 (70%)	227 (78%)		0.77	2.15	1.02, 5.55	
Private practice		947 (25%)	59 (20%)		0.52	1.69	0.77, 4.46	
**What is your highest level of education?**	4,011			0.007				0.028
Associate Degree in Nursing		2,279 (61%)	169 (58%)		—	—	—	
Bachelor of Science in Nursing		417 (11%)	47 (16%)		0.42	1.52	1.04, 2.17	
Doctor of Nursing Practice		282 (7.6%)	31 (11%)		0.37	1.45	0.94, 2.18	
Master of Science in Nursing		741 (20%)	45 (15%)		−0.13	0.87	0.60, 1.26	
**What is your employment status?**	4,011			>0.9				0.6
Contract		1,737 (47%)	133 (46%)		—	—	—	
Permanent employee		1,787 (48%)	143 (49%)		0.13	1.14	0.84, 1.54	
Visiting		195 (5.2%)	16 (5.5%)		0.21	1.23	0.67, 2.14	
**In which department do you primarily work?**	4,011			0.006				0.083
Emergency room		1,585 (43%)	157 (54%)		—	—	—	
Endocrinology		420 (11%)	23 (7.9%)		−0.49	0.61	0.38, 0.96	
General medicine		1,215 (33%)	79 (27%)		−0.34	0.71	0.49, 1.03	
Intensive care unit		279 (7.5%)	20 (6.8%)		−0.27	0.76	0.45, 1.23	
Other		220 (5.9%)	13 (4.5%)		−0.48	0.62	0.33, 1.07	
**Knowledge**	4,011			0.14				0.5
Knowledgeable		2,744 (74%)	227 (78%)		—	—	—	
Not knowledgeable		975 (26%)	65 (22%)		−0.1	0.91	0.67, 1.21	
**Attitude**	4,011			0.001				0.003
Negative attitude		621 (17%)	28 (9.6%)		—	—	—	
Positive attitude		3,098 (83%)	264 (90%)		0.6	1.81	1.22, 2.80	

### 3.6 Key observations on nurses' knowledge, attitudes, and practices

**Figure 2:**
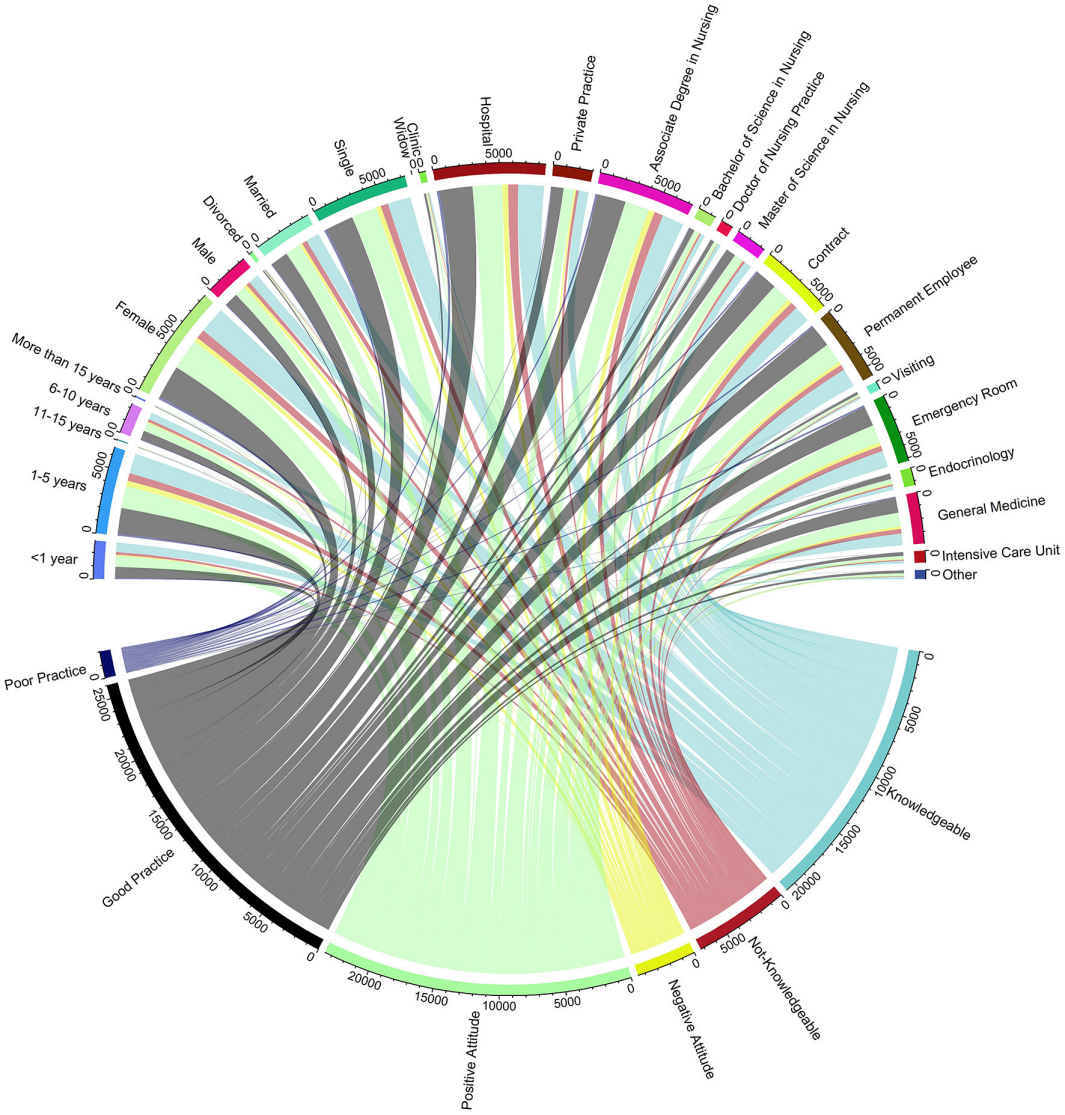
Correlation between demographic characteristics, educational background, work environment, and knowledge, attitudes, and practices. this chord diagram shows the relationships between demographic factors, educational background, work environment, and nurses' knowledge, attitudes, and practices (KAP) in diabetes management. Outer segments represent categories; inner chords indicate connections to KAP outcomes.

Nursing professionals with “1–5 years” of experience exhibit a significant inclination toward being “Knowledgeable”, evidenced by the substantial chords connecting these segments. Similarly, the “Female” gender segment has a robust association with a “Positive Attitude”, highlighted by the thick chords. In contrast, the “More than 15 years” experience category shows fewer individuals with a “Not-Knowledgeable” attribute, suggesting that extensive experience might correlate with a higher likelihood of possessing substantial professional knowledge. Educational levels, particularly those holding an “Associate Degree in Nursing”, demonstrate a strong connection to being “Knowledgeable”, while “Doctor of Nursing Practice” and “Master of Science in Nursing” segments are predominantly linked to “Good Practice”. This visualization suggests a correlation between advanced educational qualifications and the propensity to engage in best practice behaviors, as shown in Figure 2.

## 4 Discussion

The results from this study provided significant insights into the demographics, KAP, of nurses in diabetes management. A substantial majority of the participating nurses were women with a background in hospital settings, highlighting the need for targeted educational programs in these environments. While there was a commendable level of foundational knowledge regarding the diagnosis of DM, notable gaps were evident in specialized knowledge areas, particularly in managing diabetes in special populations and the use of advanced technologies. Attitudes toward interdisciplinary collaboration and the psychological aspects of diabetes care were generally positive, yet there was a disparity in recognizing the importance of cultural competence in dietary counseling. Practice assessments revealed strong adherence to best practices like regular blood glucose monitoring and participation in multidisciplinary meetings but also pointed out areas for improvement, such as the integration of psychological support and utilization of electronic health records. These findings underscored the critical areas where educational and policy initiatives could significantly enhance the efficacy and comprehensiveness of diabetes care delivered by nurses.

The study involved 4,011 registered nurses, predominantly female (71%) and with 1–5 years of experience (55%). This demographic profile aligns with findings from who reported a predominance of early-career female nurses in urban hospital settings. The high percentage of nurses holding an Associate Degree in Nursing (61%) is slightly above the national average reported by the American Association of Colleges of Nursing, which could reflect regional educational trends or specific recruitment policies of the hospitals involved in the study (34, 35). Our results demonstrated that a high proportion of participants (83%) are well-versed in the criteria for diagnosing DM, echoing findings from other studies that highlight strong foundational knowledge in healthcare professionals (36). However, our study revealed considerable knowledge gaps in managing DM, with only 61% confidence reported among participants. This discrepancy is notable when compared to the literature, where higher competence is often noted, possibly due to targeted training programs in these areas (37). The lower familiarity with pharmacological treatments in our study (34%) could be attributed to the rapid evolution of diabetes management protocols, which may outpace standard nursing curricula (38, 39).

Our analysis revealed that nurses with more than 15 years of experience were less likely to be knowledgeable, a finding that contrasts with the assumption that expertise correlates positively with knowledge. This may be due to the obsolescence of earlier training not aligned with modern guidelines (40, 41). In contrast, those with 1–5 years of experience showed a non-significantly higher knowledge level, possibly reflecting more recent and updated educational exposures. Marital status and work environment also significantly impacted knowledge levels, with single nurses and those in hospital settings displaying higher knowledge levels. This supports literature suggesting that dynamic hospital environments provide more continual learning opportunities compared to private practices (42, 43).

Notably, attitudes toward nursing practice showed a significant correlation with knowledge levels. Nurses with a positive attitude were twice as likely to be knowledgeable, a finding supported by research that links positive professional attitudes with enhanced engagement and proactive learning (44, 45). This underscores the importance of fostering supportive environments that cultivate positive attitudes among healthcare professionals (46, 47). Furthermore, this study significantly contributes to existing knowledge by highlighting the critical role of demographics and professional environment in nursing education, particularly in specialized areas of DM management.

Our study found that a significant majority (73%, *n* = 2,912) of nursing professionals recognize the importance of interdisciplinary collaboration in diabetes care, which is consistent with the literature that underscores the effectiveness of collaborative approaches in chronic disease management (36). Similarly, the appreciation for managing psychological aspects alongside physical health, as indicated by 78% (*n* = 3,139) of respondents, aligns with recommendations for holistic patient care (48, 49). However, some studies suggest potential barriers to integrating psychological care, primarily due to time constraints and lack of specific training, which might explain the lower consensus on holistic care approaches observed in our study (43% endorsement) (50–52). The strong belief in the impact of patient education on DM management outcomes, supported by 67% (*n* = 2,702) of our participants, echoes the findings of previous studies highlighting education as a critical component of effective diabetes management (53, 54). However, our results also reveal a gap in recognizing the importance of cultural competence in dietary counseling, with only 54% (*n* = 2,155) acknowledging its significance. This discrepancy could be attributed to varying levels of exposure to culturally diverse populations and suggests a need for enhanced training programs that emphasize cultural sensitivity in care provision (52, 54).

Our findings indicate a gap in nurse preparedness to assist with the financial aspects of diabetes care, as only 62% (*n* = 2,486) feel equipped to address these issues. Literature suggests that economic barriers significantly affect patient adherence to treatment plans, underscoring the need for comprehensive training that includes financial navigation (55, 56). Moreover, the concern about workload constraints, as expressed by 66% (*n* = 2,666) of the nurses, mirrors the broader issues within the healthcare system that limit the time available for holistic care (57, 58).

Our statistical analysis revealed significant determinants of nursing attitudes toward diabetes care. Experience, gender, marital status, and workplace setting were all influential, consistent with prior research indicating that these factors shape healthcare professionals' perceptions and practices (59, 60). Notably, our finding that nurses with more experience are more likely to have a positive attitude is supported by studies that link professional experience with enhanced competence and confidence in patient care (60). Our study contributes to the existing body of knowledge by providing contemporary data on nurses' attitudes toward various aspects of diabetes care, highlighting areas of consensus as well as aspects requiring further attention and development. The significant statistical correlates identified offer insights into the factors that influence healthcare attitudes and practices, thereby informing targeted interventions to enhance diabetes care quality.

The majority of nurses (80%, *n* = 3,222) demonstrated a robust engagement in multidisciplinary meetings for diabetes care planning. This finding is consistent with the literature, which emphasizes the importance of collaborative practices in enhancing patient outcomes (61). Additionally, 78% (*n* = 3,120) of nurses ensured proficiency in the use of glucose monitoring devices among patients, a practice pivotal for effective diabetes management (11, 62). However, our study also identified gaps in the integration of psychological support and the use of EHRs, with only 68% (*n* = 2,736) including psychological support in their management plans and 54% (*n* = 2,167) utilizing EHRs. These areas lag behind the optimal standards suggested by recent studies, which highlight the critical nature of comprehensive support systems and technological integration in chronic disease management (63, 64).

Our findings on professional tenure and educational background offer insights into practice variability. Intermediate-experienced nurses showed an increased, though statistically insignificant, adherence to good practices, a trend that diverges slightly from the significant positive correlation found in the literature (65). Additionally, the influence of educational background, particularly holding a Bachelor of Science in Nursing, significantly correlated with better practice standards, reinforcing the literature that links higher educational qualifications with improved care delivery (65, 66). This study highlights the complex influences on nursing practices and suggests targeted interventions and policies to improve diabetes care through education and technology use. It deepens the understanding of demographic and professional impacts on care efficiency, providing a basis for future research and policy enhancement.

Additionally, the reliance on self-reported data may introduce response bias, as participants might provide socially desirable answers rather than reflecting true behaviors or beliefs. While the stratified sampling method aimed to ensure a representative sample, the potential for selection bias exists, especially given the voluntary nature of participation. This could result in an overrepresentation of nurses who are more engaged or interested in diabetes care, skewing the results toward more favorable KAP outcomes. The exclusive use of online channels for survey dissemination might have excluded nurses with limited internet access or digital literacy, potentially biasing the sample toward those more comfortable with technology.

## 5 Conclusion

This comprehensive analysis of 4,011 nursing professionals provides critical insights into the factors influencing diabetes management KAP among registered nurses. Key findings highlight the significant impact of professional experience, educational attainment, and work environment on diabetes care competencies. Nurses with 1–5 years of experience tend to have higher knowledge levels, indicating that recent education aligns closely with current diabetes management standards. In contrast, those with over 15 years of experience show reduced knowledge, suggesting a gap between their training and modern clinical protocols. Educational qualifications profoundly influence outcomes; higher degrees, such as a Doctor of Nursing Practice, correlate with better knowledge and adherence to best practice guidelines, emphasizing the need for advanced education in enhancing nursing competencies. Attitudinal data reveal a strong recognition of the need for psychological support and interdisciplinary collaboration in diabetes care, yet there is variability in the acceptance of cultural competence in dietary counseling. This underscores the potential for targeted educational interventions to bridge these gaps. Overall, the study advocates for focused educational programs and systemic enhancements to optimize diabetes care and improve nurse efficacy and satisfaction in their critical roles.

## Data availability statement

The raw data supporting the conclusions of this article will be made available by the authors, without undue reservation.

## Ethics statement

Ethical approval was obtained from the Departmental Bioethical Committee of the Department of Endocrinology, the Central Hospital of Enshi Tujia, and Miao Autonomous Prefecture. Informed consent was taken from all participants before they participated in the study.

## Author contributions

LH: Conceptualization, Data curation, Formal analysis, Methodology, Project administration, Resources, Software, Visualization, Writing – original draft. WJ: Conceptualization, Data curation, Funding acquisition, Project administration, Software, Supervision, Validation, Writing – review & editing.
